# A value chain analysis of digitalizing hospital-at-home services in Finland

**DOI:** 10.1097/HMR.0000000000000467

**Published:** 2026-01-28

**Authors:** Fan Wang, Henna Härkönen, Gillian Vesty, Terhi-Maija Isakov, Petri Ahokangas, Irina Atkova, Miia Jansson

**Affiliations:** Fan Wang, PhD, is Post-doctoral researcher, Martti Ahtisaari Institute, Oulu Business School, University of Oulu, Oulu, Finland. E-mail: fan.wang@oulu.fi.; Henna Härkönen, Research Unit of Health Sciences and Technology, University of Oulu and Medical Research Center Oulu, Oulu University Hospital, University of Oulu, Oulu, Finland.; Gillian Vesty, PhD, is Professor, School of Accounting, Information Systems and Supply Chain, RMIT University, Melbourne, Australia.; Terhi-Maija Isakov, PhD, is Project researcher, Research Unit of Health Sciences and Technology, University of Oulu and Medical Research Center Oulu, Oulu University Hospital, University of Oulu, Oulu, Finland.; Petri Ahokangas, PhD, is Professor, Martti Ahtisaari Institute, Oulu Business School, University of Oulu, Oulu, Finland.; Irina Atkova, PhD, is Assistant Professor, Martti Ahtisaari Institute, Oulu Business School, University of Oulu, Oulu, Finland.; Miia Jansson, PhD, is Professor, Research Unit of Health Sciences and Technology, University of Oulu, Medical Research Center Oulu, Oulu University Hospital, University of Oulu, Oulu, Finland, and RMIT University, Melbourne, Australia.

**Keywords:** hospital-at-home, digitalization, value chain, remote, healthcare

## Abstract

**Background:**

In recent years, increasing numbers of elderly people and cuts to the health budget have led health care providers to search for alternative plans to balance the quality of care and the health budget. HaH is growing as an alternative model for hospital wards, with the emergence of its own value chain activities and digitalization as a differentiated strategy to provide cost-effective services.

**Purposes:**

The aim of this study is to explore the value of digitalization in the hospital-at-home (HaH) value chain in Finland. This qualitative study used both deductive and inductive methods to map the HaH value chain and find out how digitalization can help improve value-added activities.

**Methodology/Approach:**

In total, 25 semistructured single and paired interviews from all well-being service counties in Finland were carried out in September to October 2023.

**Findings:**

This study highlights the value of data interoperability, remote and real-time digital solutions, data analytics in enhancing coordination and efficiency, optimizing service delivery, improving patient experience, and supporting cost-effectiveness across the HaH value chain.

**Practical Implications:**

This study provides HaH leaders with an overview of what, where, and how digitalization can improve the quality of care and highlights the strategic importance of investing in digital infrastructure and leveraging digitalization to restructure costs. It also offers ICT companies actionable insights into the specific digital care needs within HaH service delivery, guiding the development of digital health solutions that align with frontline requirements and fostering cocreation in digital health and communication technologies.

The Nordic health care model is based on solidarity, giving priority to universal civil rights and the protection of minorities (Ramsdal & Bjørkquist, 2020). For the organization of health services, Finland is divided into 21 well-being services counties, with the City of Helsinki organizing the health services in its own region. The municipalities (local authorities) are responsible for financing primary, tertiary, and specialized health care, which is core to a functioning value-based health care system (Keskimaki et al., 2019). Value-based health care is a health care delivery model based on patient health outcomes (Porter, 2008). As part of taking a value-based health care approach, “well-being services” run by Finnish municipalities have statutory responsibility for delivering value-based health care services to the entire population (Kokshagina & Keränen, 2022).

Hospital-at-home (HaH) services represent a value-based care model for eligible patients preferring home treatment, particularly the elderly, palliative care recipients, and those with chronic conditions (Arsenault-Lapierre et al., 2021; Gonzalez-Jaramillo et al., 2021). Funded primarily through taxation (Keskimäki et al., 2019), HaH services are available across all well-being service counties in Finland and aim to mitigate adverse health outcomes and reduce health care costs by minimizing hospital admissions and facilitating early discharge (Casteli et al., 2020).

Ohvanainen et al., (2021) describe the referral pathways to Finland’s HaH services, which include emergency departments, hospital wards, outpatient clinics, primary care units, and health centers. HaH care encompasses both palliative and acute services, such as administration of parenteral antibiotics, blood products, and other intravenous treatments. Following HaH care, patients may be discharged, readmitted to the hospital, or referred to aged care, palliative wards, or hospice services as appropriate.

Arguably essential to providing value-based health care is a digitally transformed value chain for care delivery (Kokshagina, 2021). HaH service model has evolved from traditional in-hospital care to a more community-oriented approach. Delivering health care services outside the hospital setting requires seamless communication among diverse stakeholders—including suppliers, health care professionals (HCPs), and patients—as well as vertical integration across functional departments within a hospital in its value chain. Although the Finnish health care system exhibits high digital maturity, with advanced digital solutions implemented in other home-based services (Koivuluoma et al., 2022), the need for and extent to which digitalization can enhance HaH activities remains unclear (Denecke et al., 2023). Therefore, this study investigated the following research question: How can digitalization add value to enhance the HaH value chain activities?

## Conceptual Framework

Value chain reflects a firm’s strategic positioning and vertical integration, influencing performance through coordinated and collaborative service delivery. A value chain, as conceptualized by Porter (1985), consists of interrelated activities that add value by transforming inputs into outputs. Value chain analysis allows each service to be deconstructed into a series of individual but interconnected service segments where the potential for digitalizing value-added activities can be identified (Hamdan, 2017). Analyzing each step of the value chain reveals how value is created and delivered, enabling systematic planning and development (Porter, 1985).

In the context of HaH, this framework helps assess how digitalization can enhance service delivery—either through differentiation (e.g., improved patient outcomes) or cost leadership (e.g., reduced staffing inefficiencies, minimized travel, and optimized remote clinical management). Value chain analysis also supports differentiation by identifying patient-valued services that improve perceived care quality. It also examines vertical integration, where functional units collaborate to create synergies and meet patient demand. This approach enables evaluation of how digital tools can interconnect value chain elements to ensure seamless coordination and information flow (Hamdan, 2017).

Porter’s value chain is a top-down managerial framework that emphasizes production logic, sequential task execution, economies of scale, and linear workflows to optimize quality and cost control, thereby sustaining competitive advantage (Ramsdal & Bjørkquist, 2020). The primary components of a value chain typically include inbound logistics, production operations, outbound logistics, marketing and sales, and postsale services. These core activities are supported by ancillary functions such as procurement, research and development, human resource management, and corporate infrastructure.

**FIGURE 1:**
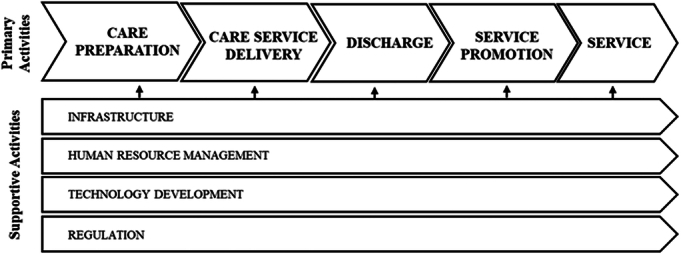
Value chain of health care delivery. Based on Porter’s (1985) framework.

Arguably, Porter’s value chain framework can inform the transition to value-based health care by distinguishing between value-adding and nonvalue-adding activities, thereby enabling more efficient resource allocation and improved patient outcomes (Porter & Teisberg, 2006). This study employs the value chain framework (Fig. 1) as a conceptual framework to analyze the role of digitalization in enhancing the value-based health care performance of HaH services in Finland. The focus of the value chain analysis is on the activities undertaken by HaH. Activity analysis is the first important step before costing the activities, using techniques such as activity-based costing, which is beyond the scope of this study (Fig. 1).

As indicated in Figure 1, the steps to health care delivery in a HaH environment would follow the generic primary and supportive activities as outlined by Porter (1995) and include primary activities from care preparation to treatment and follow-up care. The administrative support activities, such as information systems, enable a more efficient delivery of care.

## Methodology

### Research Design

This study employs a qualitative research design using content analysis to conceptualize and interpret extensive textual data (Elo & Kyngäs, 2008). To balance theoretical sensitivity with openness to emergent insights, a combined deductive and inductive analytical approach is adopted (Skjott Linneberg & Korsgaard, 2019). Deductive content analysis is used to map HaH value chain activities in Finland (Fife & Gossner, 2024). Second, an inductive approach is used to identify how and where digitalization can add value to HaH services by analyzing individual and interconnected value chain activities (Bingham & Witkowsky, 2022). This iterative process further refines the HaH value chain model by identifying the types, requirements, costs, value, and impact of digital services to be integrated.

### Data Collection

Semistructured interviews were conducted with managers in Finland’s HaH network. The doctoral researcher, with no prior relationship to participants, recruited them through telephone and e-mail using snowball sampling (Polit & Beck, 2020). To ensure national representation across all Finnish well-being service counties, participants were limited to public and private health care leaders in HaH units with comprehensive knowledge of HaH operations in their respective regions. Participants could either be interviewed individually or nominate a colleague to join the interview. Paired interviews were conducted, when possible, to achieve a shared experience from the perspective of the management of the organization providing the services. The interaction between the participants may reveal unique perspectives, which can result in deeper insight than individual interviews (Polit & Beck, 2020).

The data was collected from well-being services counties (n=21), the city of Helsinki (n=1), and private services organizations (n=3). The participants were mostly female (89%), working in middle (48%) or frontline (30%) management of public (89%) HaH services. The detailed summary of the data is shown in Table 1.

**TABLE 1:** Data collection summary

Demographics	*n* (%)
Organizational sector	
Public	41 (89)
Private	5 (11)
Gender	
Female	41 (89)
Male	5 (11)
Position	
Senior management	8 (17)
Middle management	22 (48)
Frontline management	14 (30)
Project/expert work	2 (4)

In total, 25 individual and paired semistructured interviews were conducted and recorded through Microsoft Teams in September to October 2023. The individual and paired semistructured interviews ranged in length from 58 minutes to 1 hour and 38 minutes. The interview protocol is provided in Attachment 1, Supplemental Digital Content 1, http://links.lww.com/HCMR/A181.

### Data Analysis

Automated transcripts were generated by Microsoft Teams (the protected organizational version) during interviews, which were later refined, with data analyzed and verified by the research team. The analysis began with multiple readings of the transcripts to identify open codes (unit of analysis being a thought pattern). A deductive categorization matrix (Table 2) was developed to code data aligned with the activities in the conceptual framework (Fig. 1).

**TABLE 2:** Categorization matrix by deductive analysis: HaH value chain

Predetermined Main Categories	Generic Category	Subcategory
Care preparation	Different hospital units’ coordination (emergency unit, hospital ward, outpatient unit, and different locations of HaH units)	Receiving patients-referrals Capability planning Coordinated work-regular meetings Patient registration system—data exchange between other units and the HaH
	External logistics and inventory	Delivery of individual patient supply requirements, including furniture.
	HaH work planning and coordination	Route and work planning when, where, and how often Onsite work support from doctors Digital input of patient data Understanding medical needs for care at patient's home
Care service delivery	Patient evaluation	Patient´s health condition evaluation through phone calls
	Patient-centric care	Laboratory test Infusion therapy and medication Determining appropriate follow-up actions Support from doctors in decision-making
	Patient monitoring	Patient´s health condition monitoring through phone calls
Discharge	Facilities-related outbound logistics	When to move the borrowed equipment away Returning equipment, instruments, and others back to inventory or third party
	Medicine-related outbound logistics	Medical leftovers or waste recycling
	Patient-stationery responsibility-related outbound logistics	Patient care responsibility change Sending patients to emergency or follow-up services (e.g., social work, passed away at home) Recovery at patient's home
Service promotion	Patient engagement	Brochures and materials Website
	Third-party collaboration	Collaboration with other third parties (e.g., nursing home, home care)
Service	Administration-related services	Managing bills and insurance claims Inquiry and complaints handling Patient surveys (mail, call, Internet, oral feedback)
	Care-related services	Having sufficient instructions after HaH care Scheduling appointments Follow-up check-ups
Infrastructure	Physical infrastructure	Enough workspaces Storage room for inventory Parking lots and heating for winter Medical supplies and medicines
	Virtual infrastructure	Network connection Software systems
Human resource management	Employee well-being	Salary Work safety Multitask
	Employee empowerment	Assign work according to own willingness Possibility to engage in work planning Flexibility of work Individualized working plan
	Employee support	Support/pair work Doctor availability Supervisor availability Third-party collaboration Regular meeting
	Employee training	HaH care-related training Special diseases-related training Language and cultural training
Technology development	Digital vision and strategy	Digital vision and development workshop and planning
	Digital project collaboration	Research project and research institute collaboration Pilot projects for digital services
	Digital budget	ICT budget for systems
	Digital competence training	Digital system training High-tech medical equipment training
Regulation	Procurement and data regulation	Medicine purchase law and regulation framework GDPR for data privacy and safety
	Leasing arrangements	Negotiations with leasing companies

An inductive content analysis was conducted to explore the digitalization needs within the HaH value chain. Open coding was applied to identify digitalization needs and operational gaps in interrelated activities within the HaH value chain. The resulting codes were grouped into 4 main categories where digital solutions could enhance HaH value chain performance, as shown in Table 3: (1) coordination and efficiency enhancement; (2) service delivery optimization; (3) improved patient experience; and (4) resource optimization and cost-effectiveness.

**TABLE 3:** Code structure by inductive analysis: digitalization’s potential to enhance the HaH value chain

Subcategory	Generic Category	Main Category
Availability of equipment/instruments/machines at the hospital	Coordinating and tracking logistics and inventory	Coordination and efficiency enhancement
Availability and storage of medicines at the hospital		
Care for furniture at patient's home		
Outsourced services needed for care (e.g., Leasing cars and equipment)		
Returning medical equipment, instruments, and machines		
Returning medicine back to inventory or a third party		
Availability of patient wards at different units in the hospital	Coordinating the HaH service within hospital units and social care	
Waiting time for the emergency unit		
Waiting days for receiving treatments		
Availability of HCPs at the hospital for the emergency and outpatient departments		
Social care services		
Connecting patients’ information to route planning	Coordinating HaH care planning	
Optimizing the routes for care delivery		
The possibility to know patients and their home environment in advance to prevent harm during onsite visit (e.g., mental issues) Real-time alarming system for work safety	Digital service, automation, and information	Service delivery optimization
Interoperability of data		
Digital records for medication		
Remote diagnoses from doctors for patients		
Medicine given robots and the rise of humanoid robots		
Live translation between patients and nurses		
The real-time results from laboratory testing done at the patient’s home	Clinical decision-making	
Patient’s own testing equipment		
Remote medical advice from doctors for nurses		
Multiple communication channels (e.g., chatbot, digital care pathway)	Service availability	Enhanced patient experience
Remote evaluation and check-up for patients’ conditions		
Different emotional experiences between phone calls and facial expressions in video communication	Psychological support	
Advanced delivery using drones	Reduced unnecessary visits	Resource optimization and cost-effectiveness
Remote health consultation		
Coordination and planning system	Reduced mistakes and errors	
Patient data management		
Medication data management		
Analytics and documentation		
Real-time and interoperability among systems		
Increased HaH staff satisfaction	Reduced staff retaining and training costs	
Increased patient satisfaction	Increased willingness to pay	

## Rigor and Reflexivity

To ensure the trustworthiness of this qualitative study, established criteria for qualitative research were applied (Lincoln & Guba, 1985). Credibility was supported by audio recording and verbatim transcription of interviews. Rigor was maintained through systematic data collection and analysis, with all findings thoroughly documented. Triangulation was used: 2 researchers conducted the interviews, 1 researcher performed the analysis, and 3 researchers regularly reviewed the results. Transferability was enhanced by providing a detailed contextual description. Authenticity was ensured by incorporating participants’ original expressions in citations. Data saturation was reached when no new insights emerged from additional interviews. The study adheres to the Consolidated Criteria for Reporting Qualitative Research (COREQ) checklist (Tong et al., 2007).

### Ethical Considerations

This study complies with the ethical principles of the Declaration of Helsinki. Informed verbal consent was obtained and recorded from all participants before the interviews, ensuring voluntary participation. According to Finnish legislation (Medical Research Act No. 488/1999), ethical approval was not required, as the study did not involve clinical trials, minors, or pose any physical or psychological risk to participants. Therefore, in accordance with national regulations, separate ethical committee approval was not necessary.

## Findings

**FIGURE 2:**
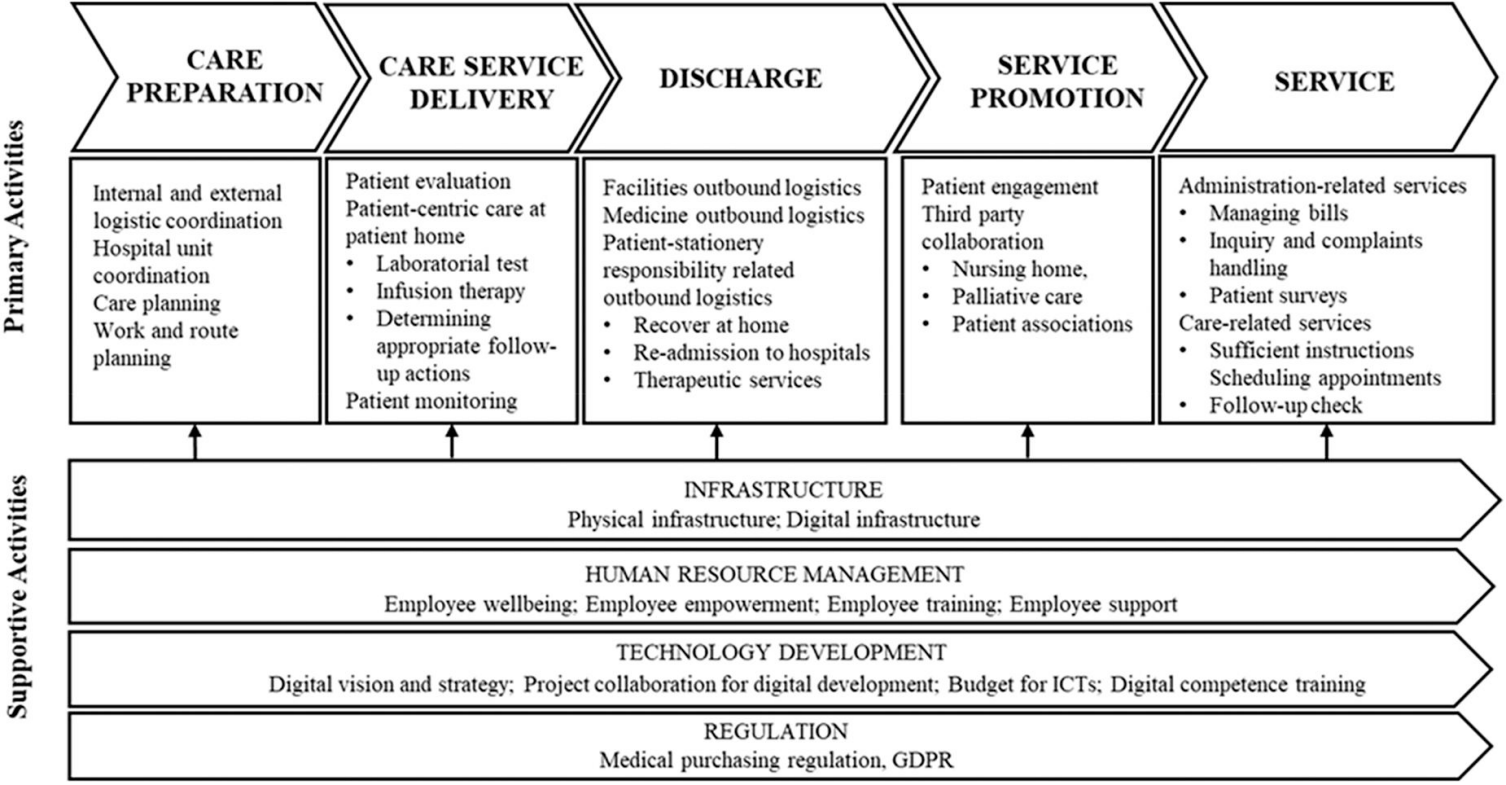
Breakdown of HaH value chain activities in the Finnish context.

A detailed breakdown of activities within Finland’s HaH value chain was mapped (Fig. 2), aligned with the conceptual framework in Figure 1, and grounded in the deductive reasoning outlined in Table 2. Figure 2 illustrates the core value chain activities underpinning HaH operations in the Finnish context. The insights into the potential and opportunities of digitalization, derived from the interviews, are presented in the findings in alignment with the HaH activity breakdown.

### Care Preparation

Interview participants highlighted the need for improved and integrated care coordination among hospital units, suppliers, and patients. Coordination typically begins with patient referrals from other hospital units; however, this process is often hindered by incomplete information, undefined terminology related to HaH services, and outdated contact details for responsible HaH nurses. These issues lead to delays and misdirected referrals, particularly when the wrong unit receives the patient. A lack of cohesion between HaH and home care services was also noted:

*The mix-up between the different services of HaH and homecare, resulting in duplicate work or wrong types of patients provided (e.g. those not able to cope at home). (Interview 22)*

Participants suggested that integrating referral information into electronic health records (EHRs) would improve coordination, replacing reliance on phone calls and paper-based communication.

The logistics for delivering care at the patients’ home requires access to motor vehicles, smart phones, tablets, and backpacks for medicines. The latter needs to be safely stored and maintained. The participants explain the importance of being aware of patients and their household conditions before onsite visits. If informed in advance of the patient’s home facilities, amenities, and treatment needs, nurses can determine the appropriate care requirements and minimize unnecessary deliveries from external logistics providers.

*It is not our task to bring care facilities, but sometimes we do take them with us (to reduce logistics) if we know the patients need them and they fit in our car, e.g., shower chairs. (Interview 3)*

A digital system coordinating logistics among suppliers and patients is essential for optimizing resource use and ensuring the timely availability of home care amenities for HaH visits. Participants also emphasized the importance of tracking medication administration and enabling real-time monitoring of resource location, quantity, maintenance, and procurement. Integration between internal and outsourced logistics enhances transparency and streamlines delivery operations.

Interviewees indicated that HaH services are delivered based on individualized work plans, which outline nurses’ schedules, visit routes, frequencies, and care activities tailored to each patient. The participant reports on the costly nonvalue-added activities around route planning that could be enhanced with digital technology:

*[It] would be more efficiently planned across the days, where to visit and whom to visit and when and what is the driving route and so on. I believe that there is a lot of waste. (Interview 6)*

Two interviewees highlighted the challenges posed by the absence of common metrics and measurements of HaH effectiveness and efficiency at both regional and national levels:

*We do not have instruments for measuring effectiveness, so we cannot provide information on the effectiveness (of HaH services). (Interview 4); I don’t think we even have tools available to calculate the potential cost savings. (Interview 5)*

Digitized data can support outcome analytics by enabling standardized metrics for evaluating the efficiency and effectiveness of HaH services. Daily digital records of care activities allow management to quantify visit volumes, assess associated costs, and define performance criteria, thereby increasing the visibility of nursing work. Value chain analysis, supported by data analytics, helps identify and eliminate nonvalue-adding activities, such as inefficient travel routes or unnecessary returns for supplies:

*If digitalization can reduce even one visit and that information can travel digitally and not by wheels of a car, then I think that is green transition. (Interview 5).*

## Care Service and Delivery

### Patient Evaluation for Admission

All interviewees emphasized the importance of staff safety in HaH services. Beyond minimizing patient-related risks in the home, participants identified nurse safety as a primary concern, particularly given that nurses often work alone in patients’ homes, including during evenings and nights. Concerns were raised about potentially unsafe living environments and their implications for staff well-being:

*Some patients, more or less, have mental problems, or, e.g., take drugs or are heavily intoxicated. To ensure work safety, we try to avoid this type of visit. (Interview 4)*

Close collaboration with home care services is essential and may involve real-time alerts or access to patient data through EHRs to assess patients’ conditions and home environments before visits; however, interoperability challenges arise due to the use of multiple standalone systems:

*Our system is not interconnected with medical and social care. (Interview 10)*

This suggests that HaH nurses currently lack access to social care and other EMR systems, which contain essential precare and social care information. To address this issue, a significant IT investment is underway in one of the well-being service counties to integrate these systems and enhance data transparency across different systems:

*Our current project is to build an integrated EHRs in our well-being service county (Interview 1).*

### Remote Versus In-Person Communication With Patients

Participants reported that telephone communication is the primary method for scheduling visits, monitoring patient conditions, and addressing emerging concerns during HaH care. However, nurses often manage multiple calls while conducting home visits, limiting their ability to respond promptly. One participant noted the burden of handling numerous patient calls alongside home visits, highlighting the need for digital solutions:

*Currently, a single staff member must handle multiple phone calls from patients, which can be quite a burden, in addition to being responsible for home visits. (Interview 7)*

Another emphasized that intermittent visual contact with end-of-life patients and caregivers could enhance their sense of safety without requiring frequent in-person visits:

*…With end-of-life care patients and their caregivers, an intermittent opportunity for discussion and visual contact could increase the feeling of safety, without the need to always visit. (Interview 23)*

Being able to see patients was considered more emotionally acceptable than impersonal phone calls, particularly in sensitive care contexts. Participants suggested using patient communication technologies—such as video calls—for remote assessment of patients and their home environments. Other participants noted that chatbots and digital care pathways can support information seeking, patient education, and continuous communication throughout the HaH care pathway.

### Care Delivery and Technology

In general, participants emphasized the need for a system that enables real-time documentation of patient medications, dosages, and usage, ensuring continuity of care across nursing staff. In a number of well-being service counties, participants reported that nurses are unable to enter patient data into EHRs during onsite visits, leading to increased workload, potential errors, and delays. This is attributed to system interoperability issues, limited network connectivity—particularly in rural areas—and occasional hardware failures. As a result, nurses often duplicate work or risk transcription errors when transferring paper records into digital systems:

*Our goal has always been to document the patient’s location, but our equipment hasn’t allowed for that yet. When we try to connect remotely to the network, the organization network simply hasn’t worked…they document it (patient data) after they go back to the hospital. (Interview 2)*

Given the 24/7 nature of most HaH services, participants also emphasized the need for real-time access to laboratory results and remote consultations to support timely clinical decision-making:

*During the weekend, drones can transport medical blood to patients’ homes and deliver blood samples from patients’ homes to hospitals without the need for an ordered taxi service. (Interview 6)*

AI can aid clinical decision-making when access to hospitals or physicians is limited, as reported by participants:

*Sometimes you can’t reach a doctor, in these cases, an AI-based clinical decision support system can be used. (Interview 2)*

Given the high demand for intravenous infusions, participants also highlighted the potential of remote monitoring and control—such as observing patients and managing infusions remotely—to reduce travel and onsite visits:

*An automatic infusion set would allow remote control of the infusion and know when to visit patient home to help. In hospitals, some have this digital control but have not yet used it for HaH services. (Interview 10)*

One participant noted that digital services could support the care of patients for therapeutic interventions:

*For some of the patients who suffer from serious pain and may be better to provide more digital therapeutic services for these patients. (Interview 9)*

## Discharge

Participants noted that HaH discharge involves transferring care responsibility to hospital units, social services, or the patient, if recovery at home is achieved. HaH nurses are responsible for evaluating patient health status, initiating appropriate referrals, and facilitating transitions to suitable care units to ensure prompt and coordinated health delivery. Participants emphasized the importance of understanding the capacity and availability of various health care units—including emergency departments, hospital wards, outpatient clinics, and HaH services across locations. This includes real-time data on outpatient wait times and bed availability for admissions. Integrated data systems were seen as essential for situational awareness and effective care coordination. One participant envisioned a map-based interface displaying the locations and activities of mobile services and professionals across sectors:

*Ideal would be a map view where you could see all mobile services or professionals whether they are rescue, emergency, homecare or home-based services…where are they moving and what are they doing. (Interview 14)*

Outbound logistics involve returning borrowed equipment from patients’ homes to inventory and managing medical waste and leftovers. This process should be closely integrated with inbound logistics, which utilizes the same system to track physical items, ensuring visibility into their availability, usage, user identity, and condition.

## Service Promotion

Marketing efforts have been undertaken to raise awareness of HaH services, aiming to engage patients and strengthen collaboration with nursing homes and patient associations:

*Not all the patients are comfortable when some strangers (nurses) are working at their home. (Interview 10)*

Participants reported that nurses primarily use printed brochures and community events (e.g., citizens’ juries, town meetings) to promote services to elder care centers and the public, with limited digital content available online. They suggested that expanding digital marketing, outlining service provision activities, could enhance value delivery to both patients and stakeholders.

## Service

Participants noted that while patients receive clear instructions for post-HaH care and follow-up appointments, it is necessary to engage staff and patients further with enhanced digital services. They emphasized the importance of supporting patients in accessing their own health data to enhance self-care using a shared platform that would facilitate patient interaction and engagement. This could also lead to expanded service provision for patients, including those who would not normally be included in HaH service provision:

*Some similar type (like diabetes patients) of health tracking could certainly be used by otherwise healthy and functional HaH clients, if connections are available for shared platforms. (Interview 4)*

In better understanding how to enhance HaH service provision, most participants reported that they use patient satisfaction surveys. However, these are primarily conducted through phone or in-person interactions. While some surveys have been mailed, they are often disregarded or not returned. Participants also noted that, unlike other well-being services, HaH receives limited online feedback, indicating a gap in digital feedback mechanisms:

*We have a (product name) feedback system on the welfare area’s website where feedback can be given. It’s very new and hasn’t started working properly. However, we do receive a lot of feedback via phone and email. We constantly get feedback from patients, but there’s no specific place to document it. (Interview 9)*

In addition to collecting feedback, participants emphasized the need for mechanisms to submit inquiries and complaints. Effective patient interaction requires a shared digital platform for 2-way communication with the HaH unit:

*A sort of direct electronic line to the HaH…for health declines…or not so acute issues…for what they have been thinking about…and there could be other information available on the same platform. (Interview 20)*

Another potential area to improve HaH service provision and communication is with language translation. It was noted that when HaH nurses encounter patients who do not speak Finnish or English, real-time interpretation tools would be valuable to ensure accurate communication and assess care needs effectively:

*We do have immigrant patients and also refugees from Ukraine, and we need to communicate and have shared understanding. (Interview 10)*

## Support Activities

### Infrastructure and Procurement

Most participants described HaH's infrastructure as fragmented, encompassing individual office locations and even nurses’ personal vehicles. They emphasized the need for integrated information systems—covering logistics, inventory, workforce planning, and patient interaction—as foundational to the HaH value chain. Particular attention was given to the need for technologically equipped, HaH-owned vehicles capable of network connectivity and real-time inventory monitoring. Medication management was noted as complex due to regulatory constraints on procurement and patient responsibility. Participants also highlighted the potential of real-time data analytics and AI to enhance service delivery, such as through immediate lab result access. Remote care requires hardware for sensing and real-time data transmission to support clinical decision-making across the care pathways.

### Technology Management and Human Resources

Participants identified patient interaction platforms as essential components of HaH infrastructure and emphasized their inclusion in ICT budgets to support digitalization. They also highlighted the value of pilot projects—particularly those in collaboration with research institutions—as an effective means of advancing digital transformation in HaH services:

*We have collaborated with XXX University on the drone testing project, and it has worked well, and hopefully funding will allow it to continue. (Interview 6)*

Participants emphasized the critical role of human resources (HR) in supporting HaH’s digital strategy, particularly through education and training. Digital competence among operational staff was reported to vary significantly, highlighting the need for attitude shifts and targeted training. Online education was suggested to build skills in supervision, collaboration, and third-party coordination, thereby supporting staff engagement and retention. Training was also deemed essential for addressing language, culture, and special needs. The remote nature of HaH work benefits from virtual platforms like Teams or Zoom, enabling real-time support from supervisors and doctors, as well as direct patient–doctor interaction through sensing and remote technologies. Access to online resources further supports nurses in delivering informed care.

### Synthesis of the Key Findings

Through value chain analysis, the findings reveal several key areas with the greatest potential for enhancing the HaH value chain. These areas are summarized in Table 4, which also outlines how the identified needs can be strategically aligned with digitalization opportunities across the HaH value chain.

**TABLE 4:** Digital solutions are enhancing HaH's value chain efficiency and communication

Inefficiency in the HaH value chain	Digital Solutions for Service Improvement
Lack of cohesion in the intraservice value chain coordination: mistakes and inconsistency	Interoperable EMR and EHR systems: integrated access to patient and hospital resource data supports administrative efficiency and vertical integration across in-hospital services, HaH, and home care Interoperable EMR and EHR systems are critical for documenting care pathways, ensuring continuity, reducing redundant testing, and facilitating seamless transitions between health care and social care units
Fragmented logistics	Real-time logistic systems: digitalize the health care supply chain, aiming to reduce delays, eliminate redundant transportation, and ensure the timely delivery of care, equipment, and medications
Lack of performance metrics and feedback analysis	Data analytics: supports the development of performance metrics, enabling the standardization of HaH services and facilitating cost-effectiveness analyses, such as historical workload, patient acuity, times of visit, and recovery date per patient, staff availability, and skill sets
Nonoptimized work and route planning	Data analytics: can analyze geographic data, patient locations, traffic patterns, and visit schedules to define the most efficient travel routes to reduce travel times and fuel costs. It can increase the number of patients seen per day
Work safety	Location tracking, emergency alerts, and real-time communication: tools enhance the safety and efficiency of HaH staff working in patients’ homes. These digital solutions support rapid response in critical situations, improve coordination, and provide staff with a greater sense of security during home visits
Challenges of health care accessibility in rural areas. HaH services are more prevalent in major Finnish cities	Remote services: to expand coverage and accessibility for patients in rural areas Data analytics: significantly enhances the efficiency of cross-county health care coordination by identifying opportunities to deliver care closer to patients, independent of administrative boundaries. This optimizes resource allocation and reduces unnecessary long-distance travel
Digital technology facilitates patient care and monitoring	Service automation: advanced technologies such as drone delivery, AI, and robotics can improve situational awareness and support clinical decision-making at the point of care. The remote patient monitoring and automated alerts (e.g., for infusion timing) support timely interventions during service delivery
Inefficiency in handling patient communication through phone calls	Patient-centered digital services: Chatbots and digital care pathways, including patient status evaluation, self-reported data collection, appointment requests, complaint handling, and feedback mechanisms, facilitate patient communication and improve access to HaH nurses, contributing to a more responsive and patient-centered care model

## Discussion

This study provides a comprehensive examination of digitalization across the HaH value chain in Finland, offering valuable insights into the digital transformation of health care services. Our findings align with Campaz-Landazábal et al., (2024) who emphasize the role of ICT in enhancing cross-level clinical coordination. It highlights the importance of digitalizing health care supply chains to generate operational benefits and support effective service delivery (Beaulieu & Bentahar, 2021). The interoperability of EMRs and EHRs has emerged as a key enabler of improved care communication and coordination (Mukhtar et al., 2023), fostering closer integration between health care and social care systems to support more patient-centric models (Heart et al., 2017).

However, these advancements are not solely technical. Regulatory and ethical challenges remain significant barriers to interoperability and data sharing (Hillebrand et al., 2023). Data protection and privacy concerns require the development of authorization frameworks and governance structures (Horn & Kerasidou, 2020). Ensuring patients have authorized access to their health information supports continuity of care and shared decision-making, enabling them to navigate services more effectively. This transparency enhances satisfaction and improves adherence to care plans (Turk et al., 2022).

Remote services are particularly valuable in improving access to care for patients in rural and underserved areas (Tong et al., 2022). They reduce unnecessary travel and enhance communication between HaH nurses and HCPs by enabling real-time observation of patients and their living environments. Service automation further supports care delivery and monitoring, reducing travel demands. Tools such as chatbots (Nadarzynski et al., 2019) and digital care pathways (Heijsters et al., 2023) improve patient communication and access to HaH nurses, contributing to a more responsive and patient-centered care model. Advanced technologies, for example, drone delivery (Scott & Scott, 2018), AI, and robotic dispensing systems (Rodriguez-Gonzalez et al., 2019) would empower nurses to make informed clinical decisions at patients’ homes through real-time access to laboratories, for example, (drone delivery), patient data, live translation, and remote guidance from physicians. Automated systems for medication administration, for example, robotic dispensing, ensure accurate documentation of dosage, timing, and type of medication, thereby preventing double dosing when multiple nurses are involved. These innovations enhance medication safety and reduce the risk of adverse drug events.

This study also highlights the role of data analytics in optimizing workflows and addressing the lack of feedback mechanisms and performance metrics in HaH services. Through data analytics, HaH leaders can identify and eliminate non-value-adding activities to optimize nurse staffing, develop relevant skill sets, align care with patient needs, and enhance satisfaction based on survey results, while simultaneously controlling costs. As noted by Mehta & Pandit (2018), data-driven insights can support standardization and facilitate cost-effectiveness analyses.

Successful digital transformation requires robust infrastructure, including a clear digital vision, dedicated ICT budgets, and coordinated working groups at both county and national levels. Investment is especially critical in rural and underserved areas, where limited network connectivity must be addressed through supportive policy (Ydyrys et al., 2023). Collaboration with universities and other stakeholders in co-developing technologies can further support this transition (Balta et al., 2021). Digitalization may also help address workforce challenges, such as nursing shortages, by optimizing workflows and enabling remote consultations. Strengthening digital competencies among HCPs is essential and requires substantial training efforts (Konttila et al., 2019).

While costing each of the activities is beyond the scope of the study, participants nonetheless commented on the potential for activity analysis to support cost savings across the value chain. The data-driven optimized routes planning reduced the nurses´ travel times, lowering personnel and material costs. The remote video consultations can reduce unnecessary in-person visits, for example, in follow-up checks. Recruiting and training qualified HaH nurses is a key human resource challenge, both time-consuming and costly. Technology improves workflow efficiency, which contributes to staff satisfaction, retention, and reduced recruitment costs—supporting the sustainable development of HaH services.

Compared with emergency and inpatient services, HaH service is generally less costly for patients and more cost-effective for health providers (Megido et al., 2023). Enhancing service visibility and accessibility through remote and online platforms increases patient satisfaction and encourages acceptance of home-based treatment. The use of multiple digital communication channels strengthens patient engagement, which can positively influence willingness to pay—2 key indicators of a health service’s cost-effectiveness (Ngorsuraches et al., 2018; Phelps, 2019). Although the focus of this study is on identifying HaH activities and associated potential for digitization, it was noted that cost-effectiveness is achieved by improving logistics, care, patient coordination, and enhancing HaH staff satisfaction and retention; and increasing patient satisfaction, willingness to pay, and service utilization.

## Conclusion

Traditionally, hospitals operate as linear value chains, beginning with patient admission and ending with discharge. HaH services disrupt this model by offering an alternative pathway that either prevents hospital admission or facilitates early discharge with continued care at home. HaH services are emerging as independent care models with their own value chains—from admission to discharge and postcare follow-up—functioning as home-based wards that extend standardized hospital care pathways. However, HaH is still often perceived as a posthospitalization service rather than a fully integrated alternative ward. Current models exhibit fragmentation across hospital units, medical supply logistics, patient home environments, and coordination with home and social care services. Moreover, technologies used within hospitals have yet to be effectively applied to HaH services. These challenges emphasize the need for digitalization to enhance integration, coordination, and efficiency.

This study is qualitative and based on a single-country context, which may limit the generalizability of its findings to other national settings. Since the interview protocol drew on existing HaH literature (Ohvanainen et al., 2021) without explicitly addressing the full value chain, some steps may be missing from Figure 2. The cost-effectiveness observed is not derived from quantified financial data, but rather from activity-based optimization and stakeholder satisfaction. Future research could use activity-based costing analysis to provide a more precise financial assessment. In addition, the current findings reflect the perspectives of HaH leaders only, which may not represent the views of HaH nurses or patients. Subsequent studies should incorporate the viewpoints of nurses and patients to better understand how digitalization has influenced service delivery and patient experience. Due to the limited number of private sector respondents in the interviews, a comparative analysis between public and private services was not feasible. Future research should consider expanding the sample to enable robust comparative studies across public and private service sectors.

## Practical Implications

This study offers health care providers valuable insights into the organization and management of HaH services in Finland, utilizing a value chain framework. It identifies key steps for the digital transformation of HaH service delivery. Health leaders face the dual challenge of reducing costs while enhancing care quality and transitioning to value-based health care, often with limited awareness of the potential of digital solutions. This study’s activity-based analysis provides a foundation for identifying where digital tools can streamline workflows and optimize service delivery. The findings highlight the strategic importance of investing in digital infrastructure and leveraging digitalization to restructure costs. Ensuring alignment between county-level and hospital ICT services is important for effective ICT budgeting and resource allocation. Moreover, attention should be paid to selecting appropriate IT partners to mitigate system fragmentation, enhance interoperability, and address cybersecurity and data privacy requirements.

This study provides ICT companies with actionable insights into specific digital care needs within service delivery. These findings can guide the development of digital health solutions aligned with frontline requirements, fostering cocreation in digital health and communication technologies. Technology implementation requires supportive structures, including dedicated ICT budgets, a clear digital vision and mission, and working groups for digital service cocreation with stakeholders. Collaboration with external stakeholders, such as universities, is also essential for piloting initiatives. Successful digital transformation depends on a cohesive infrastructure supported by both frontline HCPs and top management within a regulatory framework.

## Supplementary Material
