# Supplementary material for: A value chain analysis of digitalizing hospital-at-home services in Finland
Source: Health Care Manage Rev. 2026 Jan 28;51(2):87–98. doi: 10.1097/HMR.0000000000000467 (PMC12922686; doi:10.1097/HMR.0000000000000467)
Supplement: Supplementary file 1 [file hmr-51-087-s001.docx]

**Appendix 1.**

**Semi-structured interview themes**

1) How would you describe hospital-at-home and service models in your wellbeing services county?

2) How would you describe the impact of hospital-at-home care in your wellbeing services county?

3) How would you develop hospital-at-home care in your wellbeing services county?

4) How would you describe the cooperation and coordination between hospital-at-home and other services in your wellbeing services county?

5) How are the needs of patients and relatives taken into account in hospital-at-home care?

6) What is the current staffing situation in the hospital-at-home care?

7) What kind of space or equipment is needed to meet the hospital-at-home care needs?

8) How could digital services add value to hospital-at-home care and the patient care pathway?

9) How do you see the future of hospital-at-home care?

10) Is there anything else you would like to say concerning hospital-at-home care?

The semi-structured questions served as a framework for the study. The framework remained unchanged throughout the study.
